# Capsaicin alleviates neuronal apoptosis and schizophrenia-like behavioral abnormalities induced by early life stress

**DOI:** 10.1038/s41537-023-00406-4

**Published:** 2023-11-07

**Authors:** Shilin Xu, Keke Hao, Ying Xiong, Rui Xu, Huan Huang, Huiling Wang

**Affiliations:** 1 Department of Psychiatry, Renmin Hospital of Wuhan University, Wuhan, 430060 China; 2 Hubei Provincial Key Laboratory of Developmentally Originated Disease, Wuhan, 430071 China

**Keywords:** Schizophrenia, Schizophrenia

## Abstract

Early life stress (ELS) is associated with the later development of schizophrenia. In the rodent model, the maternal separation (MS) stress may induce neuronal apoptosis and schizophrenia-like behavior. Although the TRPV1 agonist capsaicin (CAP) has been reported to reduce apoptosis in the central nervous system, its effect in MS models is unclear. Twenty-four hours of MS of Wistar rat pups on postnatal day (PND9) was used as an ELS. Male rats in the adult stage were the subjects of the study. CAP (1 mg/kg/day) intraperitoneal injection pretreatment was undertaken before behavioral tests for 1 week and continued during the tests. Behavioral tests included open field, novel object recognition, Barnes maze test, and pre-pulse inhibition (PPI) test. MS rats showed behavioral deficits and cognitive impairments mimicking symptoms of schizophrenia compared with controls. MS decreased the expression of TRPV1 in the frontal association cortex (FrA) and in the hippocampal CA1, CA3, and dentate gyrus (DG) regions compared with the control group resulting in the increase of pro-apoptotic proteins (BAX, Caspase3, Cleaved-Caspase3) and the decrease of anti-apoptotic proteins (Bcl-2). The number of NeuN^+^+TUNEL^+^ cells increased in the MS group in the FrA, CA1, CA3, and DG compared with the control group. Neuronal and behavioral impairments of MS were reversed by treatment with CAP. Exposure to ELS may lead to increased neuronal apoptosis and impaired cognitive function with decreased TRPV1 expression in the prefrontal cortex and hippocampus in adulthood. Sustained low-dose administration of CAP improved neuronal apoptosis and cognitive function. Our results provide evidence for future clinical trials of chili peppers or CAP as dietary supplements for the reversal treatment of schizophrenia.

## Introduction

Schizophrenia is a severe mental illness with a lifetime prevalence of ~0.7% in the world^1^. It is a mental disorder characterized by positive symptoms (delusions, hallucinations, thought disorders), negative symptoms (anhedonia, social withdrawal, poverty of thought), and cognitive dysfunction^2^. Although many researchers have tried to clarify the pathogenesis of schizophrenia, the detailed mechanism is still not fully understood.

Apoptosis is one of the significant mechanisms to be explained in schizophrenia. Risk alleles related to apoptotic genes have been recently associated with schizophrenia^3^. Magnetic resonance imaging (MRI) studies have given evidence of reduced cortical gray matter volume in prefrontal temporal and parietal cortex areas, which is consistent with the postmortem evidence of reduced cortical neuropil^4^. Studies of postmortem temporal cortex in schizophrenic patients have found that Bcl-2 was reduced and the BAX/Bcl-2 ratio was increased^5,6^. Besides, some animal studies have suggested that apoptosis signaling is involved in the cellular pathology of schizophrenia^7–9^. Therefore, neuronal apoptosis is associated with the occurrence of schizophrenia.

Transient receptor potential vanilloid 1 (TRPV1) is a calcium-permeable cation channel belonging to the transient receptor potential (TRP) family of transmembrane proteins^10^. TRPV1 can be activated by temperature (>43 °C), pH (<6.0), numerous endogenous and exogenous ligands (vanilloids, cannabinoids, pro-inflammatory lipid mediators, plant and animal toxins)^11^. TRPV1 upregulation and activation mediated by pro-inflammatory mediators have brought some detrimental effects^12^. Tumor necrosis factor alpha (TNF-α) mediates inflammation in neurons through rapidly increasing sensitization and upregulation of TRPV1^13^. Interleukin-1β (IL-1β) causes anxiety in mice through TRPV1^14^. IL-1β regulates the function and activity of TRPV1 immediately through Interleukin-1 receptor type 1 (IL-1R1) combined with TRPV1^15^. Besides, interleukin-6 (IL-6) has been shown to up-regulate TRPV1 through the activation of the Janus kinase (JAK)/phosphatidylinositol3-kinase (PI3K) signaling pathway^16^. Since neuroinflammation is one of the pathogenesis of schizophrenia, this may be a possible mechanism for TRPV1 to participate in schizophrenia^17^. 8-methyl-N-geranyl-6-nonamide (capsaicin (CAP)), a major ingredient in hot peppers of the plant Capsicum genus, is a commonly used pharmacological agonist for TRPV1^18^. Many recent publications have reported the benefits of CAP in cardiovascular disease^19^, obesity^20^, cancer^21^, and the central nervous system (CNS)^22^. In the CNS, CAP has functions that reduce neuronal apoptosis. For example, CAP therapy alleviates glutamate-induced cortical neuron death in mice^23^. CAP protects against hypoxia-reoxygenation-induced apoptosis of hippocampal neurons via the PI3K/Akt-mediated signaling pathway^24^. CAP can alleviate the apoptosis rate in the cell model of Parkinson’s disease^25^. Therefore, TRPV1 may be involved in neuronal apoptosis, which could be alleviated by CAP therapy.

Early life stress (ELS) has a profound effect on brain development and is associated with schizophrenia^26^. Maternal separation (MS) is an animal model of early life stress that disrupts normal mother-child interaction and is thought to be a powerful stressor in rodents^27^. Previous studies have shown that MS can lead to schizophrenia-like behaviors in adult animals such as increased locomotor activity and pre-pulse inhibition (PPI) impairments^28^. MS can cause a decrease of neurons in male animals during adolescence^29^. Our previous study has shown that MS decreased the survival and activity of puberty-born neurons in the hippocampus and cognitive function of rats^30^. In addition, our study also showed that MS increased apoptosis of hippocampal and prefrontal neurons^31^. Therefore, the MS rat model can be used as a powerful tool to explore the neurobiological basis of schizophrenia.

At present, the effect of MS on TRPV1 expression in rat is unclear. And how CAP reduces the neuronal apoptosis in the prefrontal cortex and hippocampus and improves cognition in MS rats remains to be explored. The aim of this study is exploring the expression of TRPV1 and neuronal apoptosis in the MS model and observe the effect of CAP on the MS model.

## Methods

### Animals

Twelve male and twelve female 8-week-old Wistar rats were obtained from Beijing Vital Rival Laboratory Animal Technology Co., Ltd. Animals were mated at 3 months of age and the males were removed one week later. Mated female rats were housed individually under 12 h light and 12 h dark cycle (lights on from 8:00 a.m. to 8:00 p.m.) with free access to food and water in temperature and humidity-controlled plastic cages (22 ± 20 °C, 50 ± 10%).

### Maternal separation

MS was performed according to previous literature^30,32^. Females were checked twice a day for delivery (08:00 and 17:00) and the day of delivery was considered postnatal day (PND)0. Each pregnant rat provided an average of 10 ± 2 offspring. On PND9, about 6 litters of rats were randomly selected to MS for 24 hours. In brief, the mothers were removed at 10:00 while the pups remained at room temperature for 24 hours on PND9. On PND10, the mothers were put back in their cages at 10:00. During the period of separation, cages containing the pups were placed on heating pads maintained at 30 °C and were filled with 3 centimeters of bedding. The control group grew up naturally. All the litters were otherwise left undisturbed except for routine cage cleaning. On PND21, MS, and control pups were weaned and then group-housed by sex (3–4 per cage). Previous studies have shown that estrogen plays a key role in regulating neuronal activity and animal behavior, and alterations in estrogen signaling are linked to a range of neurological and psychiatric conditions^33,34^. One hypothesis suggests that sex differences in learning and memory may depend on gonadal hormone activity during early brain development^35^. Therefore, all subsequent experiments were carried out only on male offspring^36^.

All procedures involving animals were approved and performed according to the Institutional Animals Care Committee of Renmin Hospital of Wuhan University guidelines.

### Experimental design

Experiment 1: the effects of MS on behavior, neuronal apoptosis, TRPV1 expression in adult rats.

**Fig. 1:**
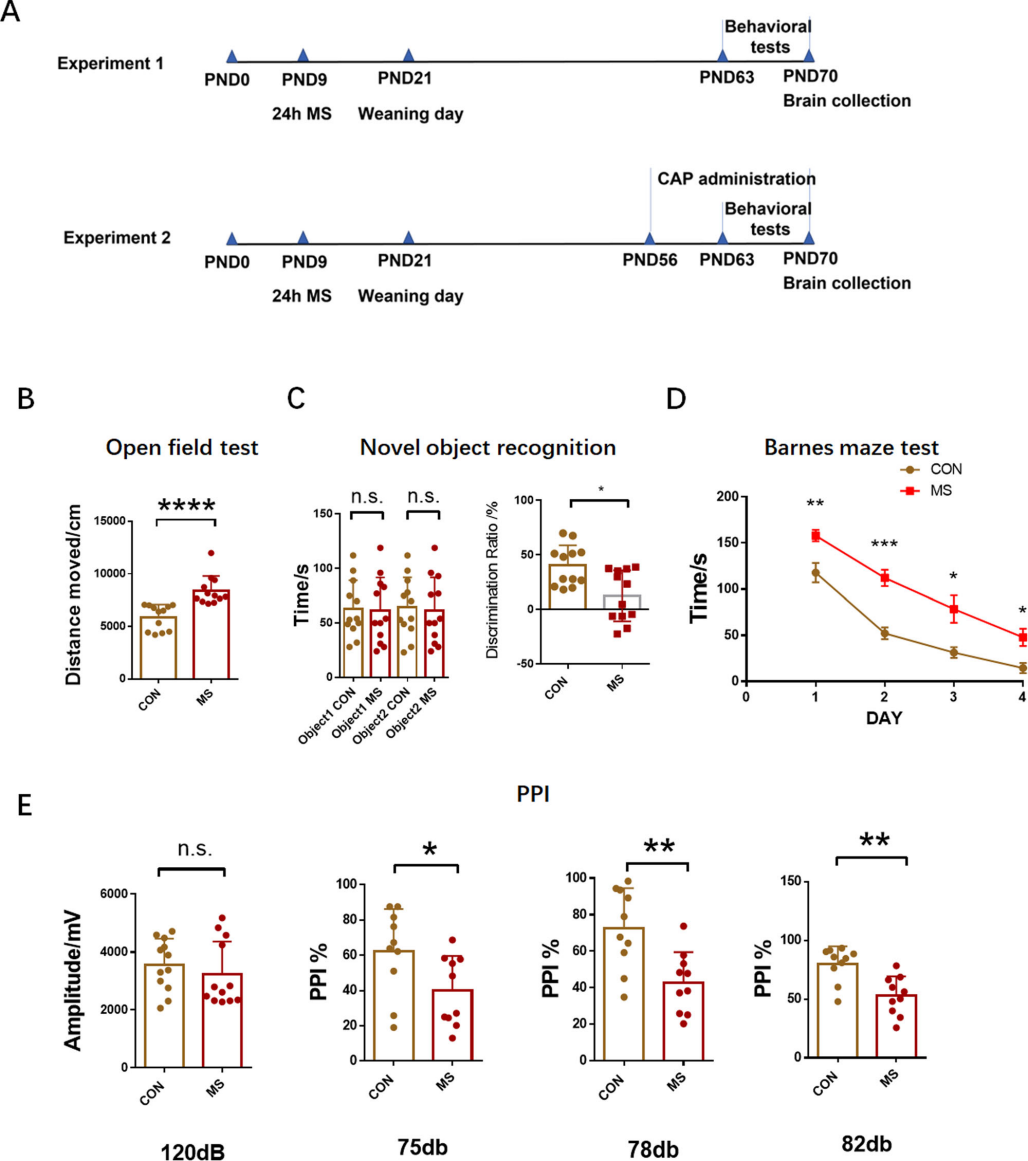
MS rats exhibit behavioral impairment in adulthood. **A** Experimental protocol. Litters were subjected to MS during lactation on PND9. Behavioral tests were performed during PND63-70. Rats received capsaicin injections intraperitoneally from PND56 to PND70. PND, postnatal day; MS, maternal separation. **B** Total distance moved in the open field in 10 minutes. **C** On day 2, time was spent exploring the two identical sample objects within the 10-min period. On day 3, time was spent exploring novel and old objects during the 10-min period. **D** The latency to the target hole in the Barnes maze test. **E** The baseline startle response to an auditory-evoked startle stimulus of 120 dB is shown in the left panel. Percentage PPI of the auditory startle reflex across different pre-pulse intensities in the right panel. CON, *n* = 12; MS, *n* = 12. n.s. not significant; *p < 0.05; **p < 0.01; ***p < 0.001; ****p < 0.0001 as compared with controls. The data are represented as mean ± SEM.

On PND9, mothers and their pups were randomly divided into control group and MS group. Control rats did not receive any treatment (normal controls), while MS rats were exposed to MS as described above. In the adult stage (PND63-70), 12 pups were randomly selected from each group and behavioral tests were conducted. After behavioral tests, animals were killed on PND70. The experimental process is shown in Fig. 1A. The remaining pups were used in experiment 2.

Experiment 2: the effects of administering CAP on behaviors, neuronal apoptosis, TRPV1 expression in the MS rats.

On PND56, each offspring group was randomly divided into two subgroups (10-12 neonates per group). From PND56 to PND70, rats were treated with either saline or CAP, and the resulting four groups were: control and vehicle group (CON + VEH), MS and vehicle group (MS + VEH), control and CAP group (CON + CAP), and MS and CAP group (MS + CAP). For the CON + VEH and MS + VEH groups, rats received vehicle (1 ml/kg/day, intraperitoneal injection) treatment, which is a 1:1:8 mixture of Tween 80: ethanol: saline. Rats in CON + CAP and MS + CAP group were subjected to CAP (1 mg/kg, intraperitoneal injection; a single injection/day) treatment for 1 week before behavioral tests and continued to be administered during the behavioral test. CAP was purchased from Sigma-Aldrich dissolved in a 1:1:8 mixture of Tween 80: ethanol: saline mixture. The experimental flowchart is shown in Fig. 1A.

### Behavioral testing of offspring

Experimental rats underwent behavioral experiments during the adult stage (PND63-70). The open field test was completed on the first day (PND63). The novel object recognition test was completed between PND63 to PND65. The Barnes maze test was completed between PND65 to PND69. On the last day (PND70), a PPI test was completed. Each rat was assessed for each behavioral measure. After each trial, the apparatus was cleaned with 75% alcohol.

### Open field test

Spontaneous motor activity was studied in a field experiment (OPF). The instrument consists of a 100 × 100 × 40 cm black plastic box with an open top. The experiment was conducted in a room with low sound and dim light (40 W, red light). A camera is fixed above the instruments and connected to monitors and video-tracking motion analysis systems (EthoVision; Noldus Information Technology in the Netherlands). Each rat was placed in the center of the open field for 10 minutes. During the observation period, the system recorded the motor activity of the rats and allowed automatic calculation of the distance the rats moved.

### Novel object recognition test

The new object recognition test was conducted using the open-field device described above and consists of three parts: habituation, training, and testing. Each rat was placed in the center of the open field to adapt to the equipment for 10 minutes. In the training experiment, the rats explored two identical objects for 10 minutes. The test experiment began 24 hours later. Rats were allowed to explore a familiar and a novel object (replacing one of the training objects) for 10 minutes. The movement was recorded by video and analyzed by EthoVision. The formula for calculating the preference for exploring novel objects is: [(time spent in the novel subject) / (total time spent in two subjects) × 100%].

### Barnes maze test

The Barnes Maze test was used to estimate spatial memory. The maze rises 140 cm above the ground, and consists of 20 round holes, each 10 cm in diameter, evenly distributed around the perimeter. The target hole is connected to a dark chamber that allows the rats to hide from bright light exposure. The day before the formal experiment, the rats were adapted to the target box for four minutes. On day 1, each rat was placed in the maze’s center black cube for 5 seconds and then explored the maze to find the target hole after the cube was removed. Once the rat entered the target hole, it was left there for 30 seconds; if it failed to find the target hole within three minutes, it was taken to the target hole and allowed to stay there for one minute. The time to the target hole was recorded. Each animal was tested twice during the day with an interval of four hours. The experiment was repeated for four days. We averaged the results of two replicates as the result for each rat on that day. The whole process was tracked by cameras.

### PPI testing

The PPI test was performed in an anechoic chamber fitted with a smaller plexiglass cage mounted on a gravity sensor platform to digitize the pressure generated by startled rats (AniLab Scientific Instruments Co., Ltd., China. www.anilab.cn.). The white noise was set at 70 dB. After 5 minutes of acclimation, 5 startle stimuli were applied (120 dB startle for 20 ms). In the testing phase, an initial delay of 50 ms was followed by a 20 ms impulse stimulus (75, 78, or 82 dB) and a 40 ms startle stimulus of 120 dB after a 100 ms delay, randomly applied by software control for about 40 trials (Supplementary 1). The interstimulus interval ranged from 10 to 30 s. The results of PPI75, PPI78, and PPI82 are calculated automatically by the system software. The percentage of PPI induced by each pre-pulse intensity was calculated as [1-(startle amplitude on pre-pulse trial)/(startle amplitude on pulse alone)]×100%.

### Sample collection

After completing behavioral tests on PND70, sodium thiopental (50 mg/kg, i.p.) was used to kill animals. Immediately after removal from the body, the prefrontal cortex and hippocampus tissues were frozen in dry ice and transferred to -80 °C for storage until further protein expression analysis was required. 3 rats in each group were injected with 4% paraformaldehyde and their brains were taken for immunofluorescence analysis or TUNEL assay. Rats were perfused through the heart initially with PBS and then with 4% paraformaldehyde. The brains were then fixed overnight in 4% paraformaldehyde, embedded in paraffin, and cut at 40 μm thickness.

### Protein extraction and western blotting analysis

Total protein was extracted from hippocampal and prefrontal tissues with RIPA buffer (Beyotime Biotech, China). Protein concentration was determined with BCA kit (P0010S, Beyotime Biotech). Identical amounts of RIPA-extracted protein were loaded and separated with 8% SDS polyacrylamide gel and transferred onto polyvinylidene fluoride (PVDF) membranes. Western blot analysis used the following primary antibodies: Mouse anti-TRPV1 (dilution 1:800, ab203103, Abcam, Cambridge, UK), rabbit anti- BAX (dilution 1:1000, GB11690, Servicebio, China), rabbit anti-Bcl-2(dilution 1:1000, abs147821, Absin, China), rabbit anti-Caspase3(dilution 1:2000, abs124299, Absin, China), rabbit anti-Cleaved-Caspase3(dilution 1:2000, abs132005, Absin, China), and rabbit anti-β-actin (dilution 1:1000, ab181602, Abcam, UK). The following secondary horseradish peroxidase (HRP)-conjugated antibodies were used at 1:5000 dilution: goat anti-rabbit HRP (12–348, Millipore, USA) and goat anti-mouse HRP (sc-2005, Santa Cruz Biotechnology, USA).

### Immunofluorescent staining assay

Rats were anesthetized and perfused through the heart initially with PBS and then with 4% paraformaldehyde. The brains were then fixed overnight in 4% paraformaldehyde, embedded in paraffin, and cut at 40μm thickness. The paraffin-embedded tissue sections were dewaxed in glycol, rehydrated, placed in sodium citrate, microwaved for antigen retrieval, washed with PBS, and then blocked in 1% BSA (Roche, Basel, Switzerland) at room temperature for 2 h. Sections were incubated with primary antibody mouse anti-TRPV1 (dilution 1:500, ab203103, Abcam, Cambridge, UK) and rabbit anti-NeuN (dilution 1:250, GB11138, Servicebio, China) overnight at 4 °C. The secondary antibody (Cy3 conjugated donkey Anti-Mouse IgG (H + L), dilution 1:200, GB21401, Servicebio, China; Alexa Fluor 488-conjugated goat Anti-Mouse IgG (H + L) dilution 1:200, GB21401, Servicebio, China) was then incubated at 37 degrees for 1 hour. DAPI (Thermo Fisher Scientific, Waltham, USA) was used as a nuclear stain. Observe and photograph with a fluorescence microscope (IX53, Olympus, Tokyo, Japan).

### TUNEL assay

Terminal-deoxynucleotidyl transferase-mediated nick end labeling (TUNEL) can be used to detect apoptotic cells. In late apoptotic cells, the sticky 3’-OH terminal is generated by the break of DNA. Under the catalysis of deoxyribonucleotide terminal transferase (TdT), dUTP with fluorescein molecule is labeled to the 3’ -terminal of DNA, and then observed by fluorescence microscope.

Paraffin-embedded tissue cross-sections were dewaxed in xylol, rehydrated, placed in sodium citrate, microwaved for antigen retrieval, and then washed with PBS and blocked with 1% BSA (Roche, Basel, Switzerland) in PBS for 2 h at room temperature. Sections were incubated at 4 °C overnight with primary antibodies targeting mouse NeuN (1:100, ab279296, Abcam, Cambridge UK), followed by incubation with Fluorescein’s TUNEL Cell Apoptosis Detection Kit (G1501-50T, Servicebio, China). Completed the experiment according to the instructions. Paraffin sections routinely deparaffinized, dehydrated with gradient ethanol, digested with proteinase K at 37 °C for 20 min, and incubated with equilibration buffer at room temperature for 10 minutes, then incubated with TUNEL mixture (TdT enzyme: FITC-12-dUTP Labeling Mix: Equilibration Buffer = 1 µL: 5 µL: 50 µL for each sample) away from light at 37 °C for 1 h. After DAPI staining the nuclear, the slides were photographed by a fluorescence microscope and analyzed for positive cells.

### Data analysis and statistics

The results are presented as the mean ± SEM. Experiment 1 was analyzed using an independent sample t-test. In Experiment 2, the data were analyzed by analysis of variance (ANOVA) to determine group differences. P values less than 0.05 (two-sided) were considered significant. All analyses were performed with GraphPad Prism software, version 7.0, or SPSS software, version 20.0.

## Results

### Effects of MS on behavior, neuron apoptosis, and trpv1 expression in rats

#### MS rats exhibit behavioral impairment in adulthood

To investigate the effects of MS on rat behavior, several behavioral tests were performed on PND70. We first evaluated the effects of MS on the locomotor activity of the rats. The open field test demonstrated that MS-treated rats had significantly increased total distances moved when compared with control rats, suggesting that the MS rats were hyperactive (CON: 5889 ± 347.2, *n* = 12; MS:8398 ± 399.0, *n* = 12; *P* < 0.0001; Fig. 1B). During the novel object recognition test, MS and control rats spent similar amounts of time exploring two identical objects during the habituation phase (Fig. 1C left panel). However, when the object2 was replaced 24 hours later, the MS group spent relatively little time exploring the new object (CON: 40.20% ± 5.397%, *n* = 12; MS: 12.59% ± 6.749%, *n* = 12; *P* = 0.0234; Fig. 1C right panel). In the Barnes maze test, from day 1 to day 4, MS rats took longer to find the target hole than controls. The results showed that MS rats developed deficits in spatial learning and memory (DAY 1: 117.9 ± 10.59 for CON *n* = 12, 158.0 ± 6.260 for MS *n* = 12, *P* = 0.0036; DAY 2: 52.28 ± 6.367 for CON *n* = 12, 112.3 ± 8.917 for MS *n* = 12, *P* = 0.0001; DAY 3: 31.34 ± 5.913 for CON n = 12,78.51 ± 14.97 for MS *n* = 12, *P* = 0.0173; DAY 4: 14.51 ± 5.509 for CON *n* = 12, 47.99 ± 9.378 for MS *n* = 12, *P* = 0.0116; Fig. 1D). In the PPI test, there was no difference in baseline response between the two groups for the 120 dB startle stimulus (Fig. 1E, left panel). However, MS rats showed impaired PPI at varying pre-pulse intensities, indicating reduced inhibition of starling stimuli compared to control rats (75 dB: 62.28 ± 7.602 for CON *n* = 12, 39.90 ± 6.258 for MS *n* = 12, *P* = 0.0355; 78 dB: 72.57 ± 6.954 for CON *n* = 12, 42.77 ± 5.279 for MS *n* = 12, *P* = 0.0068; 82 dB: 79.97 ± 4.689 for CON *n* = 12, 52.82 ± 5.185 for MS *n* = 12, *P* = 0.0021 Fig. 1E, right panel).

#### Depletion of TRPV1 in the prefrontal cortex and hippocampus of MS rats

**Fig. 2:**
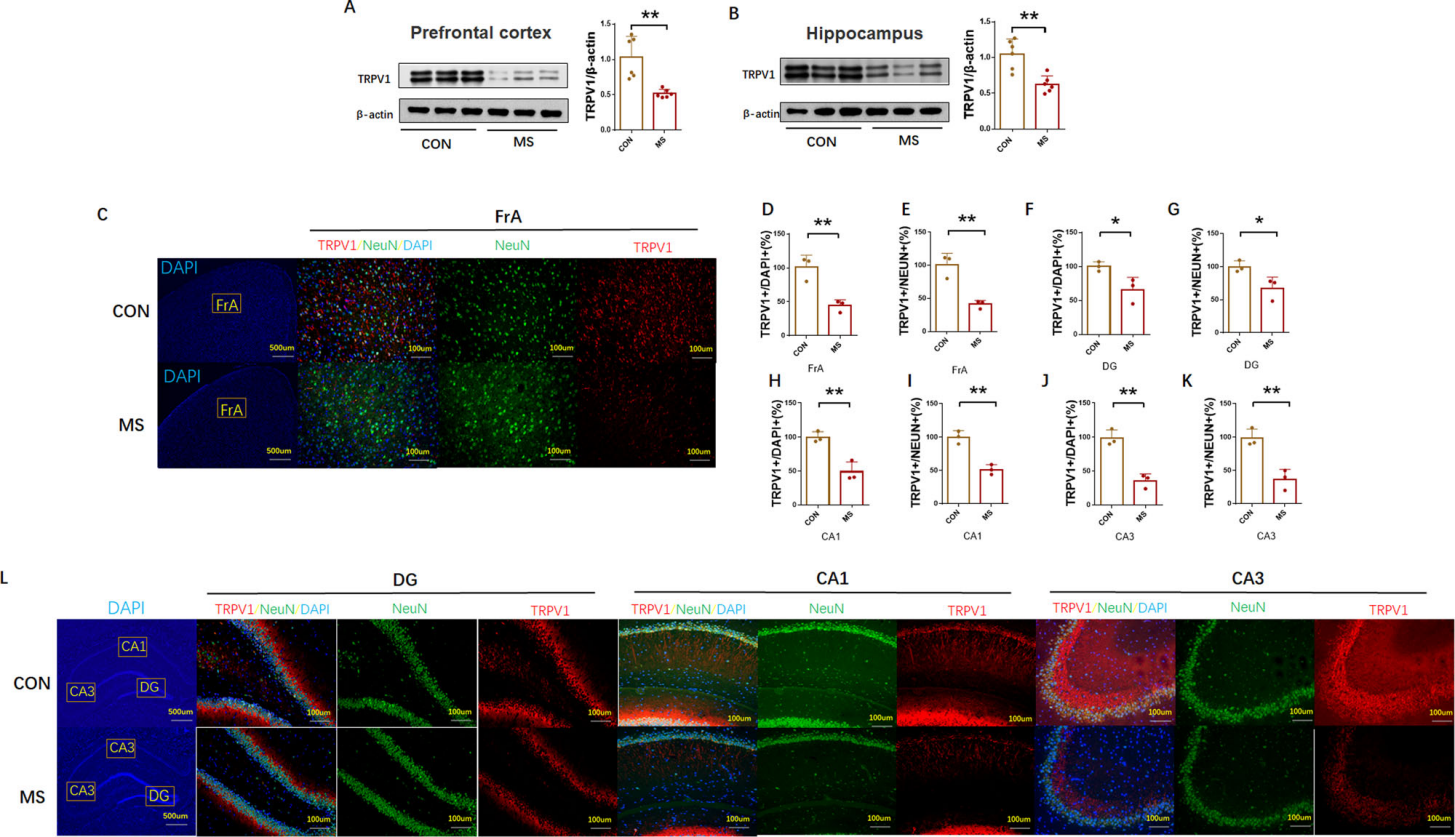
Effects of MS on TRPV1 expression in rats. **A**, **B** The protein expression of TRPV1 was measured by Western blot in the prefrontal cortex and hippocampus (*n* = 6 per group). **C**, **L** Representative immunofluorescence images show the expression of TRPV1 (red pixels), NeuN (green pixels), and DAPI (blue pixels) in the frontal association cortex (FrA) and in the hippocampal CA1, CA3, and dentate gyrus (DG) regions (*n* = 3 per group). **D**–**K** Quantitative analyses of the percentage of NeuN and TRPV1 co-labeling. Quantitative analysis of results and data are represented as mean ± SEM; *P < 0.05; **P < 0.01.

We then measured TRPV1 concentrations in adult rat brains. Western blotting results showed that TRPV1 expression levels decreased significantly in the prefrontal cortex (MS: 0.5175 ± 0.0238 relative to CON, *P* = 0.0031, *n* = 6, Fig. 2A) and hippocampus (MS: 0.6250 ± 0.048 relative to CON, *P* = 0.0014, *n* = 6, Fig. 2B) in the MS group compared with the control.

Next, a double-label immunofluorescence assay was used to detect NeuN and TRPV1 expression. As shown in Fig. 2D, F, H the number of TRPV1 positive cells (MS: 44.00% ± 5.033% relative to CON, *p* = 0.0079, *n* = 3, frontal association cortex (FrA); MS: 65.67% ±10.59% relative to CON, *P* = 0.0407, *n* = 3, dentate gyrus (DG); MS: 49.00% ±8.505% relative to CON, *P* = 0.0062, *n* = 3, CA1; MS: 35.67% ±5.897% relative to CON, *P* = 0.0022, *n* = 3, CA3) and the number of NeuN/TRPV1 co-labeled positive cells (MS: 41.00% ± 3.512% relative to CON, *p* = 0.0054, *n* = 3, FrA; MS: 67.33% ±9.684% relative to CON, *P* = 0.0407, *n* = 3, DG; MS: 51.33% ± 4.333% relative to CON, *P* = 0.0023, *n* = 3, CA1; MS: 36.67% ±8.819% relative to CON, *P* = 0.0061, *n* = 3, CA3) in the MS group decreased significantly compared with the control group.

#### Increased apoptosis in the prefrontal cortex and hippocampus in MS rats

**Fig. 3:**
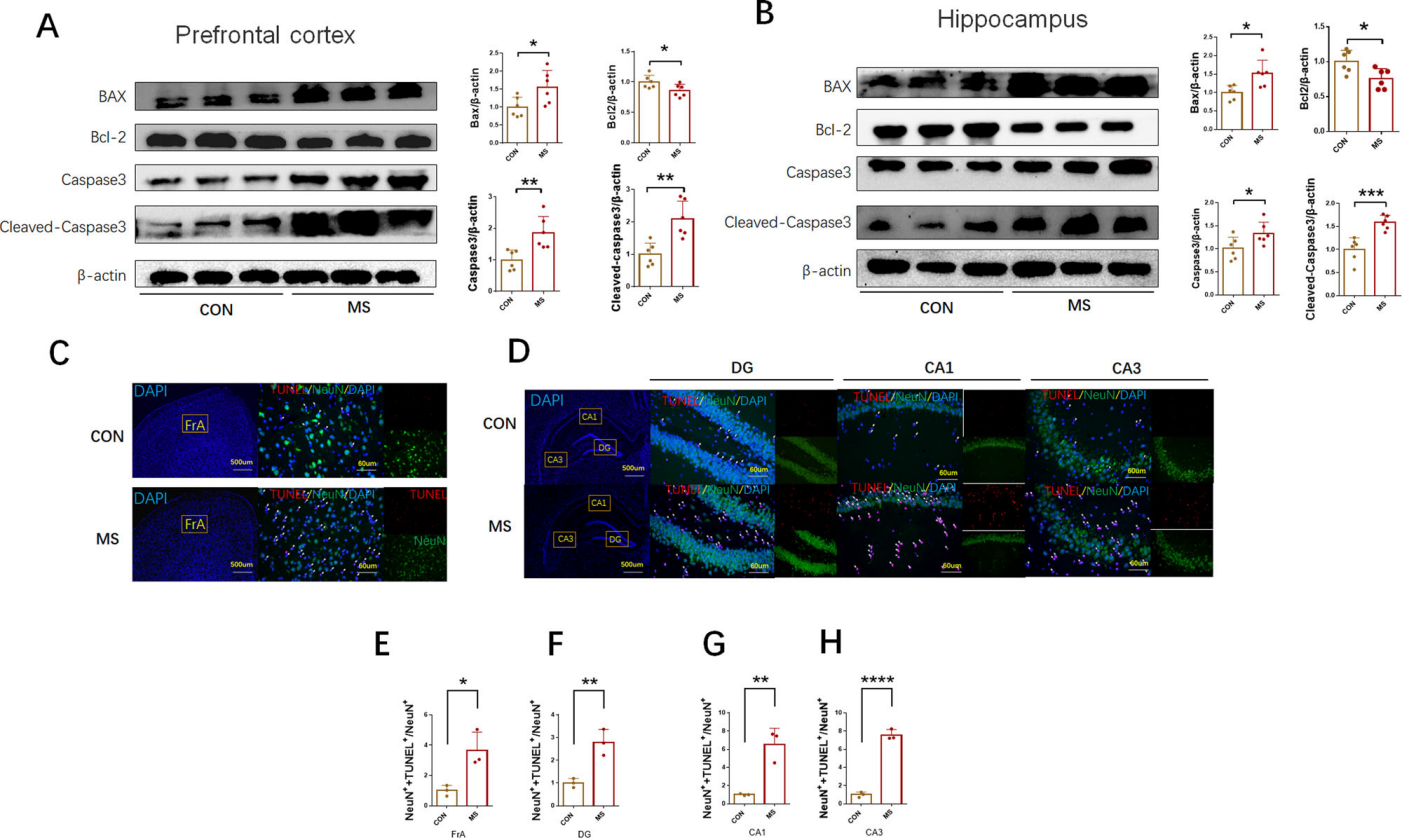
Increased apoptosis in hippocampus and prefrontal cortex in MS rats. **A**, **B** The protein expression of BAX, Bcl2, Caspase-3, and Cleaved Caspase-3 were measured by western blot in the prefrontal cortex and hippocampus (*n* = 6 per group). **C**, **D** Neuronal apoptosis was assessed by TUNEL staining in the frontal association cortex (FrA) and in the hippocampal CA1, CA3, and dentate gyrus (DG) regions. TUNEL positive cells (red), NeuN (green), and DAPI (blue) labeling (*n* = 3 per group). **E**–**H** Quantification of TUNEL‐positive cells in the FrA, CA1, CA3, and DG regions. Original magnification ×400. Data are presented as mean ± SEM for each group. *P* values were calculated using Student’s *t* test. *p < 0.05; **p < 0.01; ***p < 0.001; ****p < 0.0001.

We examined the level of apoptosis in rat brains. The expression of important apoptotic indicators (Bcl-2, BAX, Caspase-3, Cleaved Caspase-3) in the prefrontal cortex and hippocampus was detected. Pro-apoptotic protein expressions of BAX, Caspase3 and Cleaved-Caspase3 in prefrontal cortex and hippocampus were significantly increased in MS rats compared with control group (Fig. 3A, B) (prefrontal cortex: MS: 1.560 ± 0.1879 relative to CON, *P* = 0.0287, *n* = 6 BAX; MS: 1.859 ± 0.2128 relative to CON, *P* = 0.0061, *n* = 6 Caspase-3; MS: 2.088 ± 0.2280 relative to CON, *P* = 0.0023, *n* = 6 Cleaved Caspase-3) (hippocampus: MS: 1.510 ± 0.1498 relative to CON, *P* = 0.0129, *n* = 6 BAX; MS: 1.334 ± 0.1004 relative to CON, *P* = 0.0447, *n* = 6 Caspase-3; MS: 1.586 ± 0.06283 relative to CON, *P* = 0.0007, *n* = 6 Cleaved Caspase-3). However, anti-apoptotic protein expression of bcl-2 (prefrontal cortex: MS: 0.5175 ± 0.0238 relative to CON, *P* = 0.0031, *n* = 6) (hippocampus: MS: 0.7572 ± 0.0582 relative to CON, *P* = 0.0191, *n* = 6) was decreased dramatically in MS rats.

In addition, TUNEL staining was used to detect neuronal apoptosis. In PND70, compared with control group, the number of NeuN^+^+TUNEL^+^/NeuN^+^ cells in the MS group increased significantly in the FrA, CA1, CA3, and DG regions (Fig. 3C–H) (FrA: MS: 3.655 ± 0.6923 relative to CON, *P* = 0.0212, *n* = 3; DG: MS: 2.783 ± 0.3322 relative to CON, *P* = 0.0071, *n* = 3; CA1: MS: 6.540 ± 1.022 relative to CON, *P* = 0.0056, *n* = 3; CA3: MS: 7.569 ± 0.3340 relative to CON, *P* < 0.0001, *n* = 3).

### The effects of CAP administration on behaviors, neuronal apoptosis, and TRPV1 expression in the prefrontal cortex and hippocampus of MS rats

#### CAP administration improved the behavioral performances of MS rats

**Fig. 4:**
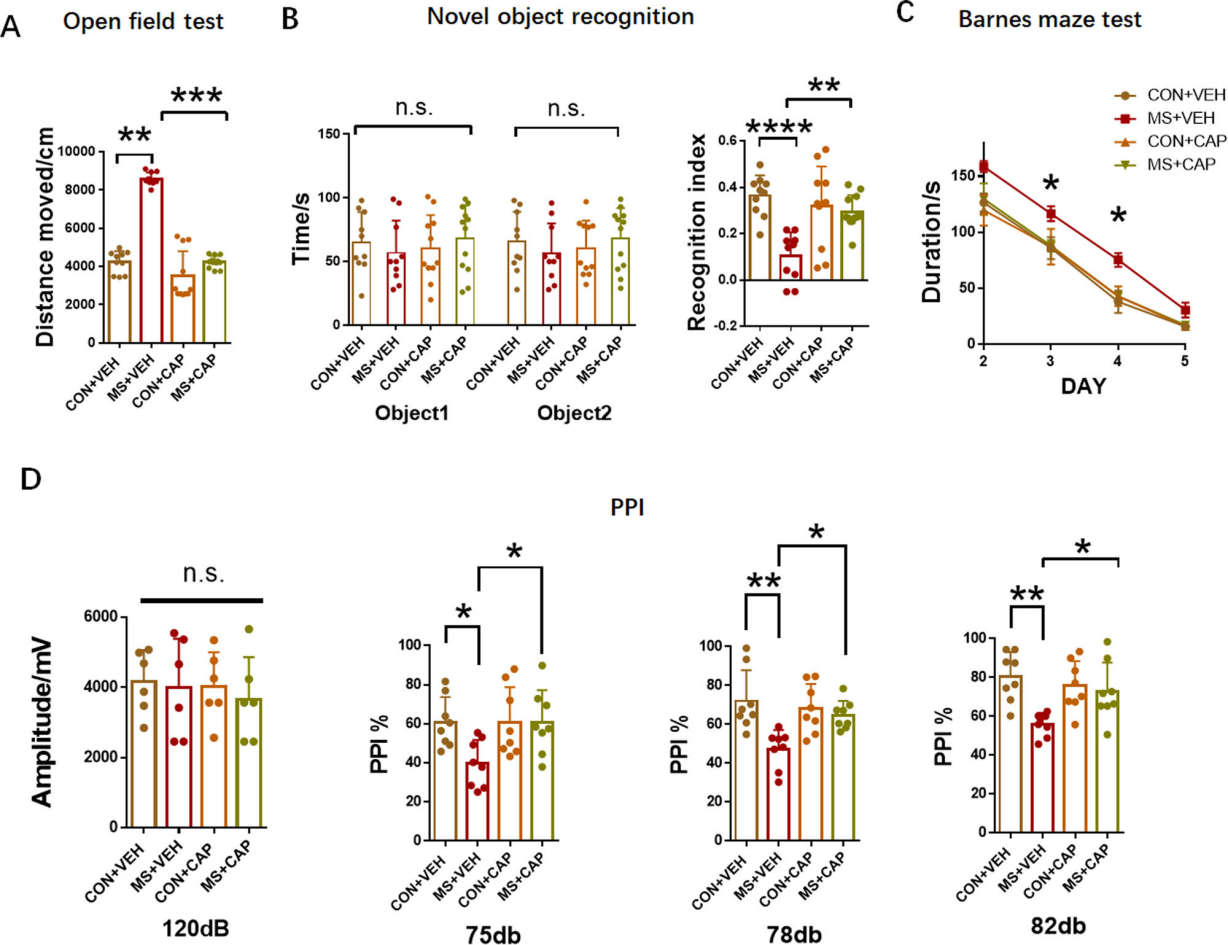
Capsaicin administration improves behavioral performances of MS rats. **A** Total distance moved in the open field in 10 minutes. **B** Effects of capsaicin administration on the novel object recognition assay in different groups. **C** Effects of capsaicin administration on the latency to the target hole in the Barnes maze test. **D** The baseline startle response to an auditory-evoked startle stimulus of 120 dB is shown in the left panel. Effects of capsaicin administration (1 mg/kg/day) on PPI at different pre-pulse intensities in the right panel. CON + VEH, *n* = 10; MS + VEH *n* = 10, CON + CAP *n* = 11, MS + CAP *n* = 12 n.s. not significant; *p < 0.05; **p < 0.0; ***p < 0.001; ****p < 0.0001 as compared with controls. The data are represented as mean ± SEM.

Behavioral tests showed that CAP 1 mg/kg/day administration improved behavior and cognition. In open field experiment, the total moved distance of MS + CAP group was lower than that of MS + VEH group (MS + VEH: 8587 ± 105.9, *n* = 10; MS + CAP: 4236 ± 91.12 *n* = 12; *P* = 0.0022, Fig. 4A). In the novel object recognition test, the MS + CAP group spent more time sniffing for the new object than MS + VEH group (MS + VEH: 0.1032 ± 0.03256, *n* = 10; MS + CAP: 0.2939 ± 0.02091 *n* = 12; *P* = 0.0023 Fig. 4B right panel). In addition, in the Barnes maze test, the MS + CAP group had decreased latency entering the target hole than the MS + VEH group (DAY 1: 158.6 ± 4.9 for MS + VEH *n* = 10, 129.6 ± 14.2 for MS + CAP *n* = 12, *P* = 0.2681; DAY 2: 116.7 ± 6.67 for MS + VEH *n* = 10, 87.93 ± 5.15 for MS + CAP *n* = 12, *P* = 0.0464; DAY 3: 75.3 ± 6.17 for MS + VEH *n* = 10, 42.8 ± 4.312 for MS + CAP *n* = 12, *P* = 0.0330; DAY 4: 30.5 ± 6.69 for MS + VEH *n* = 10, 17.1 ± 2.689 for MS + CAP *n* = 12, *P* = 0.1030; Fig. 4C). In the PPI test, there was no difference in baseline response among the four groups for the 120 dB startle stimulus (Fig. 4D, left panel). However, CAP treatment partially alleviated MS induced impaired PPI response of 75 dB, 78 dB, and 82 dB (75 dB: 39.58 ± 4.262 for MS + VEH *n* = 10, 60.71 ± 5.825 for MS + CAP *n* = 12, *P* = 0.0417; 78 dB: 47.24 ± 3.439 for MS + VEH *n* = 10, 64.77 ± 2.593 for MS + CAP *n* = 12, *P* = 0.0292; 82 dB: 55.84 ± 2.095 for MS + VEH *n* = 10, 72.56 ± 5.335 for MS + CAP *n* = 12, *P* = 0.0455) (Fig. 4D, right panel).

Side effects of CAP as pain, spasticity, and crouching were described in Supplementary 2.

#### CAP administration reversed the effects of MS on apoptosis indices but not TRPV1 expression

**Fig. 5:**
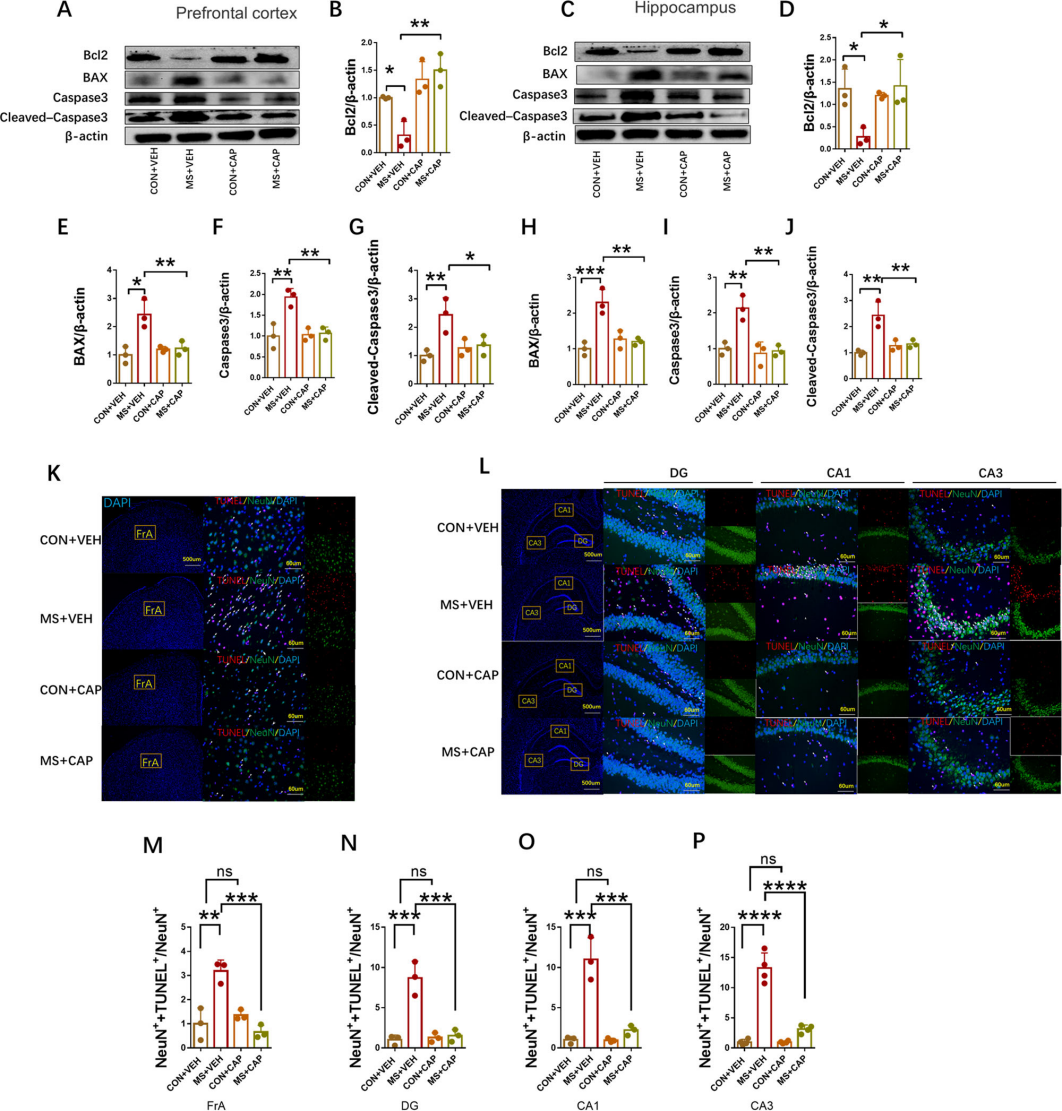
Capsaicin alleviates apoptosis induced by maternal separation in rats. **A**–**J** The protein expression of BAX, Bcl2, Caspase-3, and Cleaved Caspase-3 were measured by Western blot in the prefrontal cortex and hippocampus (*n* = 3 per group). **K**–**P** Neuronal apoptosis was assessed by TUNEL staining in the frontal association cortex (FrA) and in the hippocampal CA1, CA3, and dentate gyrus (DG) regions. TUNEL positive cells (red), NeuN (green), and DAPI (blue) labeling (*n* = 3 per group). Data are presented as mean ± SEM for each group. n.s. not significant; *p < 0.05; **p < 0.01；***p < 0.001; ****p < 0.0001 as compared with controls.

The pro-apoptotic protein expression of BAX, Caspase3 and Cleaved-Caspase3 in the prefrontal cortex and hippocampus were increased dramatically in MS + VEH compared with those in the CON + VEH group, whereas was inhibited in the MS + CAP group (prefrontal cortex: 2.433 ± 0.2963 for MS + VEH *n* = 3, 1.233 ± 0.1453 for MS + CAP *n* = 3, *P* = 0.0086, BAX; 1.933 ± 0.1202 for MS + VEH *n* = 3, 1.067 ± 0.08819 for MS + CAP *n* = 3, *P* = 0.0044, Caspase-3; 2.433 ± 0.3480 for MS + VEH *n* = 3, 1.367 ± 0.2028 for MS + CAP *n* = 3, *P* = 0.0425, Cleaved Caspase-3) (hippocampus: 2.300 ± 0.2082 for MS + VEH *n* = 3, 1.200 ± 0.05774 for MS + CAP *n* = 3, *P* = 0.0070, BAX; 2.133 ± 0.2028 for MS + VEH *n* = 3, 0.9333 ± 0.08819 for MS + CAP *n* = 3, *P* = 0.0025, Caspase-3; 2.433 ± 0.2963 for MS + VEH *n* = 3, 1.333 ± 0.08819 for MS + CAP *n* = 3, *P* = 0.0047, Cleaved Caspase-3). In addition, anti-apoptotic protein Bcl-2 expression was significantly decreased in the prefrontal cortex and hippocampus in MS + VEH treated rats, while CAP pretreatment significantly reduced these increases (prefrontal cortex: 0.3167 ± 0.1452 for MS + VEH *n* = 3, 1.500 ± 0.1732 for MS + CAP *n* = 3, *P* = 0.0020) (hippocampus: 0.2733 ± 0.1157 for MS + VEH *n* = 3, 1.417 ± 0.3420 for MS + CAP *n* = 3, *P* = 0.0273) (Fig. 5A–J). Besides, CAP administration in the MS + CAP group decreased neuronal apoptosis in the FrA, CA1, CA3, and DG regions as assessed by TUNEL staining, compared with the MS + VEH group (FrA: 3.185 ± 0.2639 for MS + VEH *n* = 3, 0.6601 ± 0.1614 for MS + CAP *n* = 3, *P* = 0.0006; DG: 8.677 ± 1.216 for MS + VEH *n* = 3, 1.539 ± 0.4332 for MS + CAP *n* = 3, *P* = 0.0003; CA1: 11.01 ± 1.542 for MS + VEH *n* = 3, 2.206 ± 0.3541 for MS + CAP *n* = 3, *P* = 0.0002; CA3: 13.26 ± 1.256 for MS + VEH *n* = 3, 3.154 ± 0.3346 for MS + CAP *n* = 3, *P* < 0.0001) (Fig. 5K–P).

**Fig. 6:**
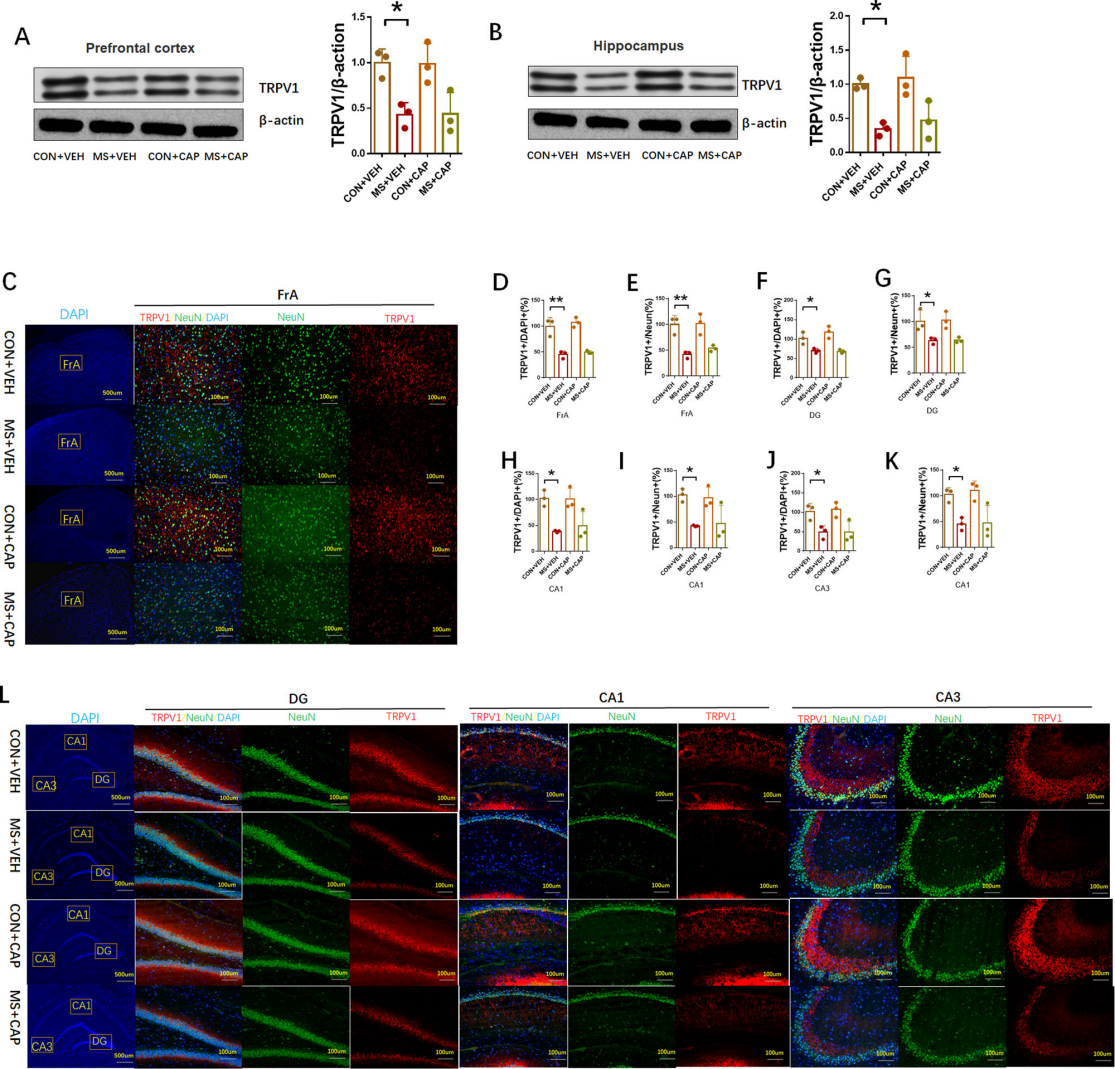
Capsaicin administration did not change TRPV1 expression. **A**, **B** The protein expression of TRPV1 was measured by Western blot in the prefrontal cortex and hippocampus (*n* = 3 per group). **C**, **L** Representative immunofluorescence images show the expression of TRPV1 (red pixels), NeuN (green pixels), and DAPI (blue pixels) in the frontal association cortex (FrA) and in the hippocampal CA1, CA3, and dentate gyrus (DG) regions (*n* = 3 per group). **D**–**K** Quantitative analyses of the percentage of NeuN and TRPV1 co-labeling. Quantitative analysis of results and data are represented as mean ± SEM, *n* = 3 for each group; *P < 0.05; **P < 0.01.

In addition, CAP administration did not increase TRPV1 expression in the MS + CAP group compared to the MS + VEH group, according to the western blotting and double-label immunofluorescence assay (Fig. 6).

## Discussion

**Fig. 7:**
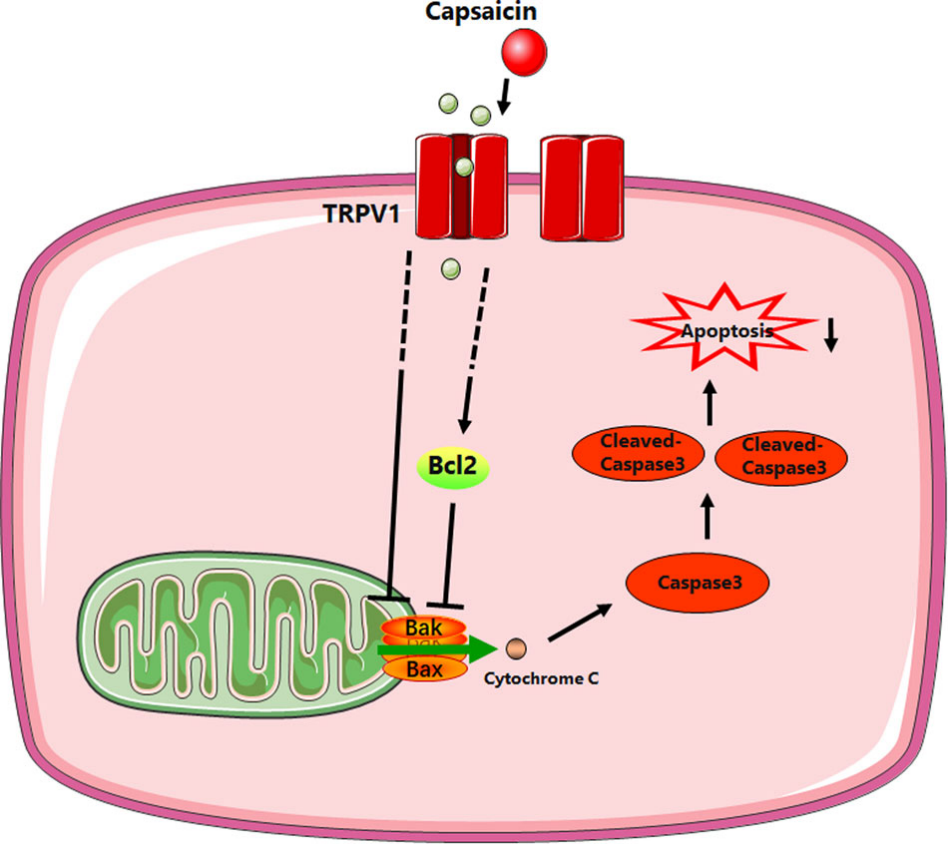
Schematic diagram of capsaicin alleviates neural apoptosis. By activating TRPV1 receptor, capsaicin increases the expression of Bcl2, and decreases the expression of BAX, which changes the permeability of the mitochondrial membrane and reduces the release of cytochrome C. Cytochrome C mediates apoptosis through activation of caspase3 and cleaved-caspase3.

In this study, we demonstrated that a single 24-hour MS in early life resulted in increased neuronal apoptosis and impaired cognitive function with decreased TRPV1 expression in the prefrontal cortex and hippocampus. And administration of the TRPV1 agonist CAP, an active component of hot peppers, significantly improved neuronal apoptosis, behavioral and cognitive function impaired by MS (Fig. 7).

The duration of separation from the mother during the first 2 years of life predicted elevated schizotypal personality disorder symptoms, which provided support for the role of early childhood psychosocial risk factors in the subsequent development of schizophrenia spectrum symptoms in emotionally vulnerable children^37^. In the study by Ellenbroek et al., the disturbance of PPI was strongest when MS was performed on PND9 compared to MS on PND3 and PND6^38^. Moreover, impairment in long-term potentiation (LTP) was evident only in adolescent animals exposed to MS on PND9, but not on PND4 and PND18^39^. Therefore, we studied in PND9 MS male rats.

Both the hippocampus and the interconnected prefrontal regions are targets that seem sensitive to early stress-induced changes^40,41^, since they mediate neuroendocrine stress responses through neuronal connections to the hypothalamic-pituitary-adrenal (HPA) axis. One hypothesis suggested that sex differences in learning and memory may depend on gonadal hormone activity during early brain development^35^. Therefore, our study focused on the prefrontal cortex and hippocampus.

There is increasing evidence that apoptosis is involved in the pathological process of schizophrenia. Many clinical studies have investigated the level of apoptosis in patients with schizophrenia^3,5,6,42^. In addition, some second-generation antipsychotics, such as risperidone paliperidone, olanzapine, and clozapine have anti-apoptotic effects^3^. It has been reported that early MS can lead to neuronal apoptosis^43,44^. Apoptotic cell death is characterized by cell shrinkage, chromatin condensation, membrane blebbing, and DNA fragmentation. Consisted with previous research, our study showed increased apoptosis in the prefrontal cortex and hippocampus in MS rats through TUNEL staining. The relative expression of Bcl-2 and BAX genes regulates apoptosis. The expression of Caspase3 and Caspase 9 genes may activate the cell death mechanism through endogenous apoptotic genes, and the expression of them in schizophrenia patients is higher than that in healthy controls^45^. In general, increased expression of BAX and decreased expression of Bcl-2 suggested apoptosis. Our study showed that MS increased BAX, Caspase3, Cleaved-Caspase3 and decreased Bcl-2. However, the specific mechanism of inducing neuronal apoptosis in schizophrenia is not fully understood.

In recent years, much literature has provided evidence that TRPV1 may play an important role in the development of schizophrenia. From a clinical perspective, pain perception deficits in schizophrenia may be related to TRPV1 changes in primary sensory neurons^46^. One of the mechanisms of cannabidiol is the activation of TRPV1. Cannabidiol can aggravate positive symptoms in patients with schizophrenia^47^. In addition, some animal studies have explored the association between TRPV1 and schizophrenia-like behavior. However, the results are not entirely consistent. Some studies have shown that TRPV1 induces or exacerbates schizophrenia-like behaviors. For example, three different indirect endocannabinoid agonists, AM404, VDM11, and AA5HT, respectively attenuated hypermotility in dopamine transporter (DAT) knockout mice, and this effect was reversed by the TRPV1 antagonist capsaepine^48^. Administration of TRPV1 agonist CAP (50 mg/kg s.c.) on day 2 of life resulted in hyperactive schizophrenia-like behavior at weeks 5–7, but increased PPI at weeks 8-12^49^. However, other studies suggested that TRPV1 activation reduces schizophrenia-like behaviors. The study conducted by Leonora E Long et al. has shown that MK-801 (0.3–1 mg/kg i.p.) dose-dependent reduced PPI inhibition and cannabidiol (5 mg/kg i.p.) successfully reversed the impairment in PPI inhibition induced by MK-801 (1 mg/kg i.p.)^50^. Results on the effects of cannabinoids on PPI in rodents indicate the involvement of TRPV1 in sensorimotor gated function and pathophysiology in schizophrenia^51^. The spontaneously hypertensive rats (SHR) showed schizophrenia-like behavior with hyperactivity and decreased sociability. TRPV1 agonist CAP (2.5 mg/kg) treatment reduced hyperactivity of SHR^51,52^.

TRPV1 is widely expressed in the nervous systems. Several studies have reported the expression of TRPV1 in neurons, astrocytes, and microglia^53^. However, its physiological roles remain controversial. TRPV1 activation in neurons increases ROS expression^54^, mitochondrial disruption, cell death^55^, mediates long-term depression^56^. However, TRPV1 activation in neurons has also been reported to protect neurons via down-regulating N-methyl-D-aspartic acid (NMDA) receptors^23^. TRPV1 activation in astrocytes secrets glutamate^57^, prolongs post synaptic excitatory^58^, mediates ER stress^59^. TRPV1 activation in microglia increases ROS^60^, promotes phagocytosis^61^, mitochondrial disruption, cytochrome C release^62^. Besides, TRPV1 activation in microglia also inhibits oxidative stress^63^, regulates microglial/macrophage M1/M2 polarization^64^, and alleviates neuroinflammation. It appears that TRPV1 activation could play a therapeutic role by stabilizing glial cells in some disease models. However, it can also cause damage under physiological conditions. This may be related to the disease model and the dose and type of agonist.

Previous studies have reported that CAP, as an activator of TRPV1, improved cognition and behavior. CAP treatment daily significantly can improve spatial learning, memory and synaptic function in Aβ42-treated Alzheimer’s disease mice^65^. CAP reduced the brain’s Aβ content and reversed cognitive decline in Alzheimer’s model mice^66^. Furthermore, a diet rich in CAP may have beneficial effects on AD cognitive function in middle-aged and elderly adults^67^. TRPV1 activation by CAP enhances NMDAR-dependent CA1 LTP and ameliorates stress-induced memory decline, which can be blocked by TRPV1 antagonists capsazepine and SB366791^68^. Consistent with previous results, our study also showed that the use of TRPV1 agonists improved cognition and other schizophrenia-like behavior. However, other studies have come to different conclusions. Repeated oral administration of CAP increases anxiety-like behaviors with prolonged stress response in rats^69^. Epileptic activity was increased in hippocampal slices of rats by CAP administration^70^. Therefore, we consider that the different effects caused by CAP may be related to the use of different disease models. The dose was given in reference to Jeong Yeob Baek’s study^71^.

Some studies have reported that CAP plays a protective role in the central nervous system. For example, in a rat model of the middle cerebral artery occlusion/reperfusion, peri-infarct injection of CAP (1 or 3 nmol) reduced infarct volume and improved neurological function^23^. Intraperitoneal CAP can reduce neurodegeneration in the substantia nigra in the lipopolysaccharide (LPS)-lesioned inflammatory rat model of Parkinson’s disease^72^. Our study found that CAP (1 mg/kg, intraperitoneal injection for 1 week), as an activator of TRPV1, could decrease the apoptosis of neuron, inhibit the expression of BAX, Caspase3, and Cleaved-Caspase3 and increase the expression of anti-apoptotic protein Bcl-2 in the hippocampus and prefrontal cortex of MS rats, which might demonstrate the anti-apoptotic effect of CAP in our model of MS. Furthermore, CAP 1 mg/kg/day intraperitoneal injection mediated paroxysm, spasticity and couching behavior last for 1–2 min only at 1st day, and did not cause death in rats, which is considered as a small and safe dose^71^.

Our study suggested that CAP administration might improve cognitive function and behavior by inhibiting neuronal apoptosis at least in MS models. However, this study still has some limitations. Because only total cell extract levels were measured, it was not possible to infer how much TRPV1 was indeed expressed and fully functional in the membrane. In addition, we only studied the expression of TRPV1 in neurons, the expression of TRPV1 in microglia and astrocytes still needs to be studied in the future. Because TRPV1 expression was decreased in MS group compared to control, we used TRPV1 agonists CAP, but not detect the activation of TRPV1, although many research have reported TRPV1 was activated by CAP^73,74^. Furthermore, we only studied male rats in order to eliminate the additional effects of estrogen, future work needs to explore the effects in females and possible sex differences.

## Conclusions

This study suggested that exposure to MS may lead to increased neuronal apoptosis and impaired cognitive function with decreased TRPV1 expression in the prefrontal cortex and hippocampus in adulthood. Sustained low-dose administration of CAP improved neuronal apoptosis and cognitive function. CAP has potential advantages as an intervention strategy for schizophrenia as a natural component of spicy foods. Our study provided new information on the etiology of schizophrenia. Our results provided evidence for future clinical trials of chili peppers or CAP as dietary supplements for the therapy of schizophrenia.

## Availability of data and material

Data will be made available on request.

### Supplementary information

SUPPLEMENTAL MATERIAL
