# Supplementary material for: Capsaicin alleviates neuronal apoptosis and schizophrenia-like behavioral abnormalities induced by early life stress
Source: Schizophrenia (Heidelb). 2023 Nov 7;9(1):77. doi: 10.1038/s41537-023-00406-4 (PMC10630396; doi:10.1038/s41537-023-00406-4)
Supplement: Supplementary file 1 — SUPPLEMENTAL MATERIAL [file 41537_2023_406_MOESM1_ESM.docx]

**
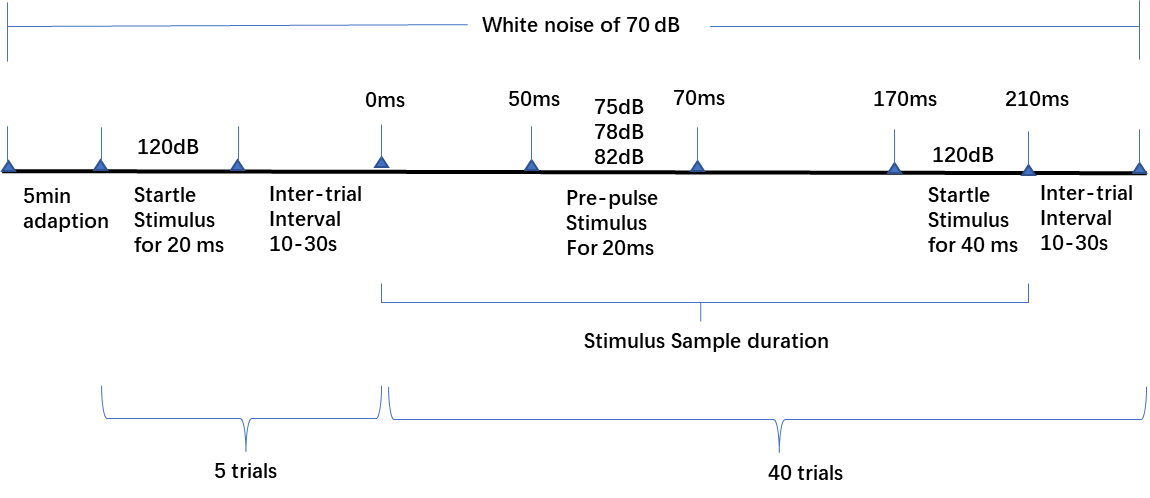
Supplementary 1. Line graph depicting the stimulus protocol to assess the sensorimotor gating in PPI test.**

**Side effects of capsaicin administration**

| last time | pain  n (%) | spasticity  n (%) | crouching  n (%) | dead  n (%) |
| --- | --- | --- | --- | --- |
| <1minutes | 10 (43.5%) | 7 (30.4%) | 11 (47.8%) | 0 (0%) |
| 1-2minutes | 13 (56.5%) | 2 (8.7%) | 7 (30.4%) |  |
| >2minutes | 0 (0%) | 0 (0%) | 0 (0%) |  |

All the side effects occurred only on the first day.

**Supplementary 2.** **Side effects of capsaicin administration.**
